# The role of mitofilin in left ventricular hypertrophy in hemodialysis patients

**DOI:** 10.1080/0886022X.2018.1456455

**Published:** 2018-04-05

**Authors:** Qi-Shun Wu, Qing He, Jian-Qiang He, Jun Chao, Wen-Yan Wang, Yan Zhou, Ji-Zhuang Lou, Wei Kong, Jun-Feng Chen

**Affiliations:** a Division of Nephrology, Affiliated Hospital of Jiangsu University, Jiangsu University, Zhenjiang, China;; b Division of Hemodialysis, Nanjing First Hospital, Nanjing Medical University, Nanjing, China;; c Division of Nephrology, The Third Affiliated Hospital of Nanjing University of Chinese Medicine, Nanjing, China

**Keywords:** hemodialysis, left ventricular hypertrophy, peripheral blood mononuclear cells, mitochondria, mitofilin

## Abstract

Left ventricular hypertrophy (LVH) is a common abnormality in hemodialysis (HD) patients. Mitochondrial dysfunction contributes to the progression of LVH. As an inner mitochondrial membrane structural protein, mitofilin plays a key role in maintaining mitochondrial structure and function. The aim of this study was to investigate the relationship between mitofilin and LVH in HD patients. A total of 98 HD patients and 32 healthy controls were included in the study. Serum N-terminal proBNP (NT-proBNP), endothelin-1 (ET-1), and atrial natriuretic peptide (ANP) were examined. The protein level of mitofilin and the mitochondrial DNA (mtDNA) copy number were estimated in peripheral blood mononuclear cells (PBMCs). The left ventricle mass index (LVMI) was evaluated in all participants, and the interaction between these variables and the LVMI was assessed. The LVMI was positively correlated with the NT-proBNP, ET-1, and ANP levels, and it was negatively correlated with mtDNA copy number and mitofilin levels. Multiple regression analysis showed that the NT-proBNP, ET-1, and ANP levels as well as mitofilin levels and mtDNA copy number were associated with the LVMI. Although further research of these associations is needed, this result suggests that LVH may affect the levels of mitofilin in HD patients.

## Introduction

Cardiovascular diseases (CVDs), including coronary artery disease, congestive heart failure, and sudden cardiac death, are the main cause of morbidity and mortality in hemodialysis (HD) patients. Left ventricular hypertrophy (LVH) is a major cardiovascular risk factor that predicts mortality in HD patients [1,2]. Hypertension, diabetes, increased body mass index (BMI), gender, age, anemia, and hyperparathyroidism have been described as the risk factors for LVH [3]. However, other risk factors may be involved in the pathophysiological process, and they must be investigated.

Mitochondrial dysfunction, which commonly presents in patients with end-stage renal disease (ESRD), contributes to LVH [4,5]. Mitofilin, a mitochondrial inner membrane protein that is highly expressed in the heart [6], plays a central role in maintaining cristae morphology and structure [7]. Experimental studies have shown that mitofilin is an important factor implicated in cell apoptosis, oxidative stress, and mitochondrial biogenesis [8–10]. However, its impact on LVH in dialysis patients has been unclear until now.

To clarify whether mitofilin is associated with LVH in HD patients, the expression of mitofilin was evaluated in the peripheral blood mononuclear cells (PBMCs) of ESRD patients undergoing HD.

## Methods

### Study design

Ninety-eight patients undergoing HD treatment at the Affiliated Hospital of Jiangsu University (Zhenjiang city, Jiangsu province, China) and Division of Hemodialysis of Nanjing First Hospital (Nanjing city, Jiangsu province, China) were enrolled in this cross-sectional study. The control group included 32 healthy controls. The following inclusion criteria were applied: at least 18 years old, with at least 1 year of HD treatment, three times per week, with each session lasting for 4 h. The following exclusion criteria were applied: malignancies, acute infections, diabetes, autoimmune diseases and a history of atrial fibrillation, myocardial infarction or heart failure with ejection fraction (EF) < 50%. The control group included healthy individuals who were at least 18 years old, without a known disease who were not taking any medications. Any patient medications that affected LVH, such as erythropoietin, angiotensin receptor blockers (ARB), angiotensin-converting enzyme inhibitors (ACEI), β-blockers and calcium channel blockers, were recorded. The study protocol was approved by the Local Ethics Committee of Affiliated Hospital of Jiangsu University and Nanjing First Hospital. All patients provided signed informed consent, in accordance with the Declaration of Helsinki principles.

### Echocardiography

Two-dimensional, M-mode, transthoracic, color Doppler echocardiography was conducted for all participants. For all participants, echocardiography was performed by a single trained cardiologist, who was blinded to the patients’ clinical records. Standard M-mode measurements of the left ventricular diastolic dimension (LVDd), left ventricular systolic dimension (LVDs), left ventricular diastolic posterior wall thickness (LVPWT), and interventricular septal thickness (IVST) were measured. Left ventricular mass (LVM) was calculated using Devereux’s method [11]: LVM (g) = 1.04 × [(LVDd + IVST + LVPWT)^3^ – LVDd^3^] –13.6. Body surface area (BSA) was also measured for each participant, and left ventricle mass index (LVMI) was calculated by LVM/BSA. LVH was defined as LVMI ≥135 g/m^2^ for men and as ≥110 g/m^2^ for women.

### Clinical and biochemical measurements

Patient demographics and clinical data, including age, gender, BMI and medications, were collected at the time of enrollment. The mean systolic blood pressure (SBP) and diastolic blood pressure (DBP) were monitored at home.

Blood samples were collected immediately prior to the start of HD. Laboratory assessments were performed for routine hematology (platelet count and hemoglobin) and serum biochemical parameters, including calcium, phosphorus, alkaline phosphatase, albumin, parathyroid hormone (PTH), C-reactive protein (CRP), triglyceride, total cholesterol, N-terminal proBNP (NT-proBNP), endothelin-1 (ET-1), and atrial natriuretic peptide (ANP). NT-proBNP was examined using high-sensitivity, noncompetitive radioimmunoassay. Plasma ET-1 and ANP levels were determined using radioimmunoassay techniques.

### PBMC isolation

The PBMCs were isolated from whole blood by Ficoll-Hypaque gradient centrifugation [12]. They were washed three times in phosphate-buffered saline (PBS), and were counted at a concentration of 10^6^/ml. The cells were collected for the next examination.

### Mitochondrial DNA (mtDNA) copy number

Total DNA in PBMCs was extracted using the DNeasy tissue kit (Qiagen, Valencia, CA), according to the manufacturer’s instructions. Reactions were performed in an ABI PRISM 7300 real-time PCR system (Applied Biosystems, CA). mtDNA, forward (5′-3′) CGATTCTTT-ACCTTTCACTTCAT CTT, reverse (5′-3′)GAGGGCGTCTT-TGATTGTGT; 18 s rRNA forward (5′-3′) GCGGTTCTA TTTTGTTGGTTTT, reverse (5′-3′) ACCT CCGA.

CTTTCGTTCTTG.18S rRNA served as controls for mtDNA for reaction efficiency. The results were analyzed using the comparative cycle threshold (ΔΔCt) method.

### The measurement of mitofilin

Proteins in the PBMCs were separated using sodium dodecyl sulfate polyacrylamide gel electrophoresis and transferred to a PVDF membrane (Millipore, MA). The membranes, which were washed with skim milk, were incubated with primary antibodies against mitofilin (Abcam, UK) and GAPDH (Santa Cruz Biotechnology, Santa Cruz, CA, USA), followed by peroxidase-conjugated secondary antibodies at room temperature. Finally, the proteins were detected by an ECL advanced system (GE Healthcare, Chalfont St. Giles, UK).

### Statistical analysis

SPSS for Windows version 18.0 was used for the statistical analysis. Data about continuous variables were expressed as the mean ± SD, and non-normally distributed variables were expressed as medians (interquartile ranges). Intergroup comparisons were analyzed with Student’s *t*-test or Mann–Whitney *U*-test. The chi-squared test was used for categorical variables. In the univariate analysis, covariates of *p* < .1 were then examined by multiple regression analysis, which was performed to describe the relationship between the LVMI and variables with *p* < .05 between groups. A *p* value < .05 was considered to be significantly different.

## Results

### Patient characteristics

The clinical and biochemical parameters are presented in Table 1. Of 98 HD patients included in the study group mean age, 51 ± 10 years, 27 were female and 71 were male. The two groups did not differ in terms of age, gender, and BMI.

**Table 1.** Demographic and clinical characteristics of the HD patients and control group.

	Control group (*n* = 32)	HD patients (*n* = 98)	*p*
Age (years)	44 ± 8	51 ± 10	.282
Gender (male, %)	24 (75)	71 (72)	.136
BMI (kg/m^2^)	21.2 ± 1.9	22.3 ± 2.7	.745
Dialysis vintage (months)	–	44.7 ± 11.0	–
SBP (mmHg)	115.4 ± 9.8	137.8 ± 12.9	<.001
DBP (mmHg)	72.0 ± 6.8	79.7 ± 9.5	.126
Kt/V	–	1.68 ± 0.45	–
URR	–	0.74 ± 0.06	–
Hemoglobin (g/L)	152.8 ± 10.1	114.5 ± 8.2	<.001
Platelet count (10^9^/L)	165.0 (129.0–219.0)	150.0 (125.0–205.5)	.115
Albumin (g/L)	45.6 ± 2.6	41.8 ± 4.2	.256
TC (mmol/L)	4.6 ± 0.5	4.4 ± 0.8	.682
TG (mmol/L)	2.5 ± 0.8	2.6 ± 0.6	.145
ALP (u/L)	84.6 ± 10.5	66.9 ± 13.5	.207
Calcium (mmol/L)	2.3 ± 0.6	2.2 ± 0.9	.145
Phosphate (mmol/L)	1.6 ± 0.4	1.6 ± 0.8	.329
PTH (pg/mL)	41.5 (25.3–55.8)	244.6 (99.7–341.2)	<.001
CRP (mg/L)	4.5 (2.3–6.0)	2.0(1.0–5.0)	.388
LVMI	75.6 ± 10.4	132.6 ± 15.8	<.001

HD: hemodialysis; BMI: body mass index; SBP: systolic blood pressure; DBP: diastolic blood pressure; URR: urea clearance rate; TC: total cholesterol; TG: triglyceride; ALP: alkaline phosphatase; PTH: parathyroid hormone; CRP: C-reactive protein; LVMI: left ventricular mass index.

### Biochemical and echocardiographic findings

**Figure 1.**
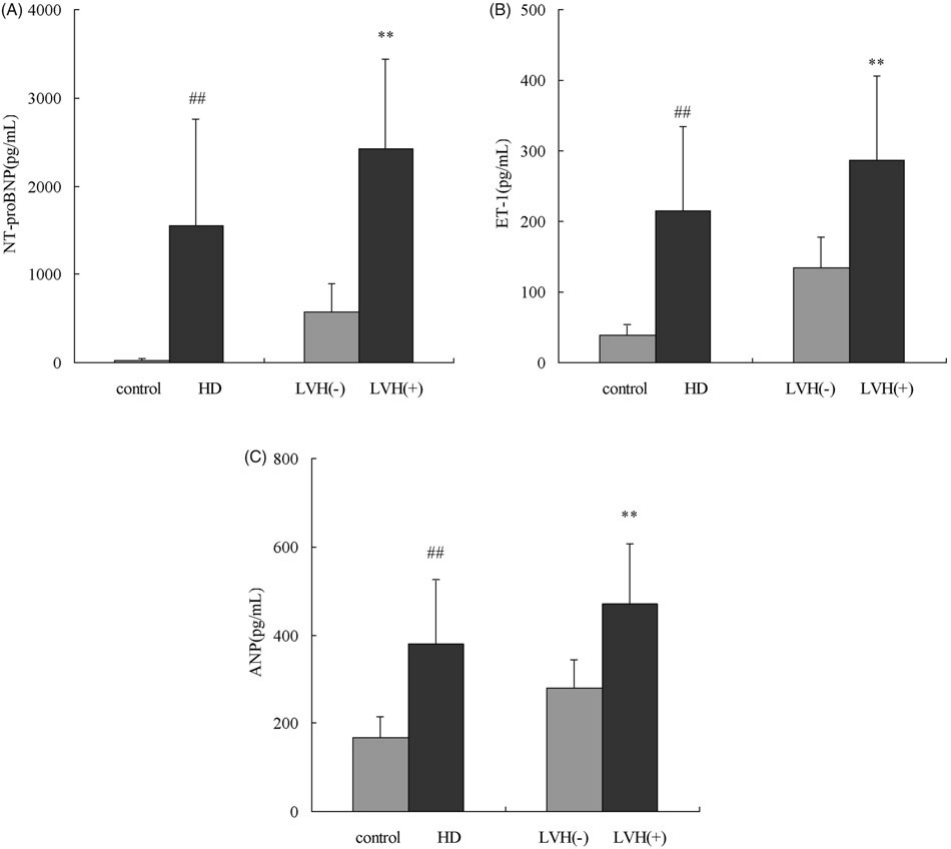
Cardiac marker levels in the HD patients and control group. NT-proBNP: N-terminal proBNP; ET-1: endothelin-1; ANP: atrial natriuretic peptide; LVH: left ventricular hypertrophy. ##*p* < .01 vs. control group; **p < .01 vs. patients without LVH.

HD patients were divided into two groups according to the presence of LVH. Fifty-two patients had LVH, but the others had findings that were within normal limits. The demographics and clinical properties of the two groups are presented in Table 2. The two groups were homogenous in terms of age, gender, BMI, urea reduction ratio (URR), kt/V, hemoglobin, blood platelet, CRP, albumin, calcium, phosphorous, PTH, total cholesterol, triglyceride, and alkaline phosphatase. The LVH patients had an LVMI of 144.8 ± 27.2 g/m^2^, while the patients without LVH had an LVMI of 108.8 ± 10.2 g/m^2^. Dialysis vintage was longer in the LVH group compared with the non-LVH group (52.8 ± 12.9 vs. 36.9 ± 10.4 years; *p* *<* .001, respectively). As shown in Figure 1, the difference in NT-proBNP between the two groups was statistically significant (*p* < .01). ANP was significantly higher in the LVH group compared with the non-LVH group (*p* < .01). The ET-1 level was also significantly higher in the LVH group compared with the non-LVH group (*p* < .01).

**Table 2.** Demographic and clinical parameters of HD patients with and without LVH.

	LVH (–) (*n* = 46)	LVH (+) (*n* = 52)	*p*
Age (years)	49 ± 12	53 ± 14	.422
≤40	10	10	
41–59	22	24	.946
≥60	14	18	
Gender (male, %)	30 (65)	41 (79)	.132
BMI (kg/m^2^)	20.8 ± 3.9	23.3 ± 4.4	.476
Dialysis vintage (months)	36.9 ± 10.4	52.8 ± 12.9	<.001
SBP (mmHg)	131.6 ± 12.5	143.3 ± 10.8	.335
DBP (mmHg)	76.6 ± 4.8	83.3 ± 10.6	.242
Kt/V	1.72 ± 0.68	1.66 ± 0.29	.521
URR	0.78 ± 0.09	0.70 ± 0.04	.889
Hemoglobin (g/L)	118.0 ± 10.7	111.5 ± 7.8	.266
Platelet count (10^9^/L)	162.0 (124.0–230.0)	136.5 (124.3–204.8)	.546
Albumin (g/L)	42.9 ± 3.8	40.5 ± 2.2	.452
TC (mmol/L)	4.8 ± 0.4	4.2 ± 0.6	.420
TG (mmol/L)	2.0 ± 0.6	2.8 ± 0.9	.361
ALP (u/L)	59.8 ± 14.3	78.5 ± 11.6	.095
Calcium (mmol/L)	2.2 ± 0.4	2.1 ± 0.8	.465
Phosphate (mmol/L)	1.5 ± 0.7	1.8 ± 0.4	.066
PTH (pg/mL)	262.7 (87.7–379.4)	233.1 (133.2–320.7)	.081
CRP (mg/L)	2.0 (1.0–5.0)	3.0 (1.0–6.0)	.760
LVMI	108.8 ± 10.2	144.8 ± 27.2	<.001
Erythropoietin (*n*, %)	18 (39%)	24 (46%)	.483
ACEI or ARB (*n*, %)	20 (43%)	25 (48%)	.648
β-blockers (*n*, %)	11 (24%)	16 (31%)	.448
Ca^2+^ channel blockers (*n*, %)	17 (37%)	23 (44%)	.465

BMI: body mass index; SBP: systolic blood pressure; DBP: diastolic blood pressure; URR: urea clearance rate; TC: total cholesterol; TG: triglyceride; ALP: alkaline phosphatase; PTH: parathyroid hormone; CRP: C-reactive protein; LVMI: left ventricular mass index; ACEI: angiotensin-converting enzyme inhibitors; ARB: angiotensin receptor blockers.

### mtDNA copy number and mitofilin in PBMCs

**Figure 2.**
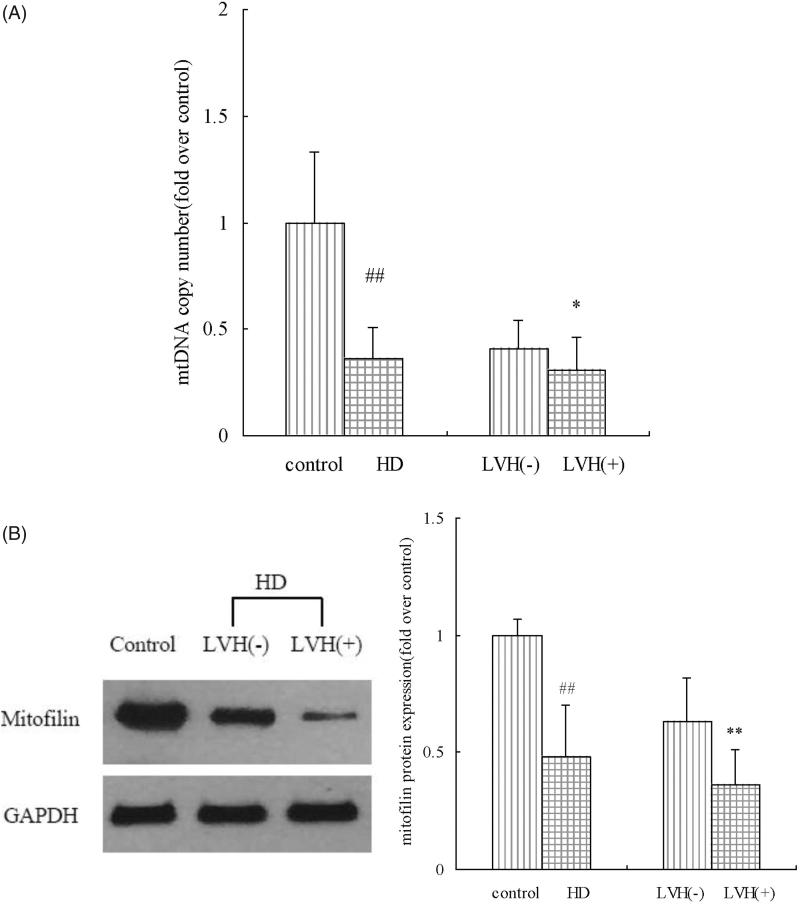
mtDNA copy numbers and mitofilin protein levels in PBMCs from the control and HD groups. HD: hemodialysis; LVH: left ventricular hypertrophy; mtDNA: mitochondrial DNA; PBMCs: peripheral blood mononuclear cells; ##*p* < .01 vs. control group; *p < .05 vs. patients without LVH; **p < .01 vs. patients without LVH.

The mtDNA copy number measured through quantitative PCR was significantly lower in the HD patients compared with the healthy subjects (*p* < .01), and a significant difference was observed between the patients with and without LVH (*p* < .05). In the LVH patients undergoing HD, the expression level of the mitofilin protein was significantly lower (*p* < .01) (Figure 2).

### Association of LVH with outcomes

LVMI was negatively correlated with the mtDNA copy number and mitofilin expression, and there was a significant correlation among NT-proBNP, ET-1, ANP, and LVMI in the HD patients (Table 3). To determine whether any of these factors were independent predictors of LVH, multiple linear regression analyses were performed, indicating that the mtDNA copy number (*p* < .05), mitofilin expression (*p* < .01), NT-proBNP (*p* < .05), ET-1 (*p* < .05), and ANP (*p* < .05) were significantly correlated with the LVMI (Table 3).

**Table 3.** Univariate and multiple linear regression analyzes of the LVMI and variables in HD patients.

Variables	*R*	*p*
Age	–0.298	.567
BMI	–0.025	.757
Dialysis vintage	0.214	.268
SBP	–0.016	.568
DBP	–0.056	.729
Kt/V	–0.178	.297
URR	–0.142	.462
Hemoglobin	–0.056	.689
Platelet count	–0.012	.676
Albumin	–0.085	.826
TC	–0.148	.398
TG	–0.240	.082
ALP	–0.089	.326
Calcium	–0.336	.088
Phosphate	0.152	.329
PTH	–0.012	.828
CRP	–0.160	.621
NT-proBNP	0.526	<.001
ET-1	0.420	.001
ANP	0.587	<.001
mtDNA copy number	–0.322	.001
Mitofilin	–0.589	<.001
Variables	*β*	*p*
NT-proBNP	0.115	.022
ET-1	0.188	.039
ANP	0.278	.034
mtDNA copy number	–0.228	.028
Mitofilin	–0.313	.007

Adjusted *R*^2^ = 0.599, *p* < .001. The independent variables used for multiple regression analysis were selected by univariate linear regression analysis (*p* < .1).

HD: hemodialysis; BMI: body mass index; SBP: systolic blood pressure; DBP: diastolic blood pressure; URR: urea clearance rate; TC: total cholesterol; TG: triglyceride; ALP: alkaline phosphatase; PTH: parathyroid hormone; CRP: C-reactive protein; NT-proBNP: N-terminal proBNP; ET-1: endothelin-1; ANP: atrial natriuretic peptide; mtDNA: mitochondrial DNA; LVMI: left ventricular mass index.

## Discussion

In this study, we demonstrated that mitofilin, a previously identified mitochondrial protein expressed in PBMCs, may be correlated with LVH in HD patients. This study is the first to reveal the relationships between the mitofilin expression of PBMCs and LVH in HD patients.

In terms of dialysis vintage, there was a significant difference between the patients with and without LVH. In a retrospective case-control study, dialysis vintage was significantly longer in HD patients with LVH [13]. LVH is present in 68–89% of incident HD patients, and it is frequently progressive, although regression is observed in a minority of patients [1].

Cardiac biomarkers, including NT-proBNP, ET-1, and ANP, are often elevated in HD patients demonstrating the presence of LVH. In this study, NT-proBNP, ET-1, and ANP levels were significantly higher in HD patients with LVH compared with patients without LVH. These biomarkers were positively correlated with LVMI, and were independently associated with LVMI. In a study by Lee et al. [14], log NT-proBNP demonstrated an independent relationship with LVMI in 44 patients undergoing maintenance HD. A study of peritoneal dialysis (PD) showed that ET-1 was an independent predictor of cardiac valve calcification at baseline and is associated with LVH [15]. In a study of 112 dialysis patients with no clinical evidence of congestive heart failure, plasma ANP levels were correlated with LVMI and inversely correlated with left ventricular EF [16]. In our study, these biomarkers served as an illustration to demonstrate the importance of LVH in dialysis patients with ESRD.

Mitochondrial dysfunction increases the risk of CVD and mitochondria, associated with left ventricular dilation, play a role in LVH [17]. mtDNA damage and depletion have not been implicated in the process of pathologic cardiac remodeling [18]. In this study, healthy subjects had a significantly higher mtDNA copy number than the patients undergoing HD, and a significant difference was observed between the HD patients with and without LVH. Similarly, Rao et al. showed that the mtDNA copy number was lower among older dialysis patients compared with older healthy subjects, and a one-log increase in the mtDNA copy number was associated with a decreased risk of mortality [19]. Another study has also indicated that the mtDNA copy number decreased as the severity of CKD increased [20]. More large-scale studies are needed to determine whether maintaining the mtDNA copy number ameliorates LVH in dialysis patients.

Mitofilin proteins are crucial organizers of mitochondrial architecture [7], and the knockdown of mitofilin in HeLa cells with RNAi led to the fragmentation of the mitochondrial network and disorganization of the cristae [10]. Our study found that mitofilin levels decreased significantly in HD patients compared with healthy volunteers, and there was a significant relationship between mitofilin levels in PBMCs and LVMI in HD patients. In a transgenic diabetic mice overexpressing mitofilin, cardiac contractile dysfunction was attenuated [9]. In another murine model, MA-5 interacting with mitofilin improved the respiration of cardiac cells [21]. There have been few studies of mitofilin in CKD patients, and more research is needed to investigate the relationship between mitofilin and LVH.

This study has some limitations. The enrolled cohort size was small. Larger studies are needed to demonstrate convincing significant differences. Further prospective studies are needed to confirm the correlation between LVH and mitofilin expression in cardiomyocytes in the progression of CVD in HD patients.

## Conclusions

This study showed that mitofilin expression in PBMCs is associated with LVH, possibly indicating that to prevent LVH in HD patients. It is beneficial to maintain mitochondrial function and mitofilin levels.
